# Cellular and functional insights into FIH-mediated hydroxylation of TRPA1

**DOI:** 10.1016/j.jbc.2025.110882

**Published:** 2025-11-04

**Authors:** Tao Guo, Dianne Marquez Lopez, Siyuan Wang, Liudi Yao, Xi Li, Elizabeth R. Davies, Mariana Vargas-Caballero, Nullin Divecha, Christopher J. Schofield, Katrin Deinhardt, Yihua Wang

**Affiliations:** 1 School of Biological Sciences, Faculty of Environmental and Life Sciences, University of Southampton, Southampton, UK; 2 UK Dementia Research Institute at The University of Edinburgh, Centre for Discovery Brain Sciences, Edinburgh, UK; 3 Clinical and Experimental Sciences, Faculty of Medicine, University of Southampton, Southampton, UK; 4 Institute for Life Sciences, University of Southampton, Southampton, UK; 5 Department of Chemistry and the Ineos Oxford Institute for Antimicrobial Research, Chemistry Research Laboratory, University of Oxford, Oxford, UK; 6 Department of Cell Biology, University of Bremen, Bremen, Germany; 7 NIHR Southampton Biomedical Research Centre, University Hospital Southampton, Southampton, UK

**Keywords:** transient receptor potential channel, ankyrin, hypoxia-inducible factor, factor-inhibiting HIF, post-translational modification, hydroxylation

## Abstract

Transient receptor potential cation channel, subfamily A, member 1 (TRPA1), also known as transient receptor potential ankyrin 1, is an ion channel located on the plasma membrane of cells. It is best known as a sensor for pain, cold, and itch in humans and other mammals, as well as for detecting electrophilic sensory irritants, including allyl isothiocyanate. A previous study confirmed that TRPA1 undergoes hydroxylation at Asn336, catalyzed by the 2-oxoglutarate oxygenase factor–inhibiting hypoxia-inducible factor (FIH). However, the biological significance of this modification remains unclear. Here, we present cellular and functional studies on the consequences of FIH-mediated asparaginyl hydroxylation of TRPA1. Coimmunoprecipitation experiments indicate that TRPA1 interacts with FIH in cells, in a manner likely involving the FIH dimer interface, as demonstrated by studies with the L340R FIH variant, which is unable to dimerize. Functional studies suggest that FIH-mediated hydroxylation may be linked to allyl isothiocyanate–induced channel activation. This response is diminished or delayed in TRPA1-expressing human embryonic kidney 293T cells and absent in primary hippocampal cultures when FIH activity is lacking. These findings highlight a potential new avenue for the therapeutic manipulation of TRPA1.

Transient receptor potential cation channel, subfamily A, member 1 (TRPA1), also known as transient receptor potential ankyrin 1, is a member of the transient receptor potential channel family (1). As a nonselective cation channel that is predominantly permeable to calcium (Ca^2+^), TRPA1 can be activated by a number of reactive and nonreactive compounds and is thus considered a “chemosensor.” In addition to sensory neurons (2), TRPA1 is expressed in hippocampal and cortical neurons (3), where it alters electrophysiological properties of action potentials (4). Allyl isothiocyanate (AITC) potently activates TRPA1 (5) *via* covalent modification of a key cysteine residue within its N-terminal cytosolic domain (6).

Evidence has been reported suggesting that TRPA1 may function in a manner related to oxygen/hypoxia sensing (7, 8). TRPA1 channel activity has been reported to be regulated by the catalytic activity of the hypoxia-inducible factor (HIF)–α prolyl hydroxylases (7). However, the prolyl hydroxylation of TRPA1 in cells requires further validation, as a subsequent study has shown a lack of activity of recombinant prolyl hydroxylase domain protein 1 to 3 on reported non-HIF substrates, including TRPA1 (9). By contrast, Saward *et al.* have shown that factor-inhibiting HIF (FIH) can catalyze hydroxylation of TRPA1 at asparagine (Asn, N) 336 within an ankyrin repeat domain (ARD) (10), although the biological significance of this modification remains unclear.

FIH, encoded by the *HIF1AN* gene, is an Fe (II)- and 2-oxoglutarate-dependent dioxygenase, which acts as a negative regulator of HIF activity. FIH regulates HIF activity *via* hydroxylating a conserved Asn residue within the C-terminal activation domain of HIF-1/2α, a post-translational modification that blocks interactions between the HIFα-C-terminal activation domain and CBP/p300 (11, 12, 13, 14, 15, 16, 17). FIH has multiple non-HIF substrates, including members of the ARD protein family, consistent with roles beyond those of HIF regulation (18, 19, 20, 21, 22, 23, 24). Either *via* HIF-dependent and/or HIF-independent pathways, FIH has been reported to regulate metabolism (25, 26, 27, 28, 29), keratinocyte differentiation (30), vascular endothelial cell survival (31), lung tissue stiffness (32), tumor growth (33, 34, 35) and metastasis (36), as well as Wnt signaling (37).

Here, we present cellular and functional investigations on the role of FIH-catalyzed hydroxylation of TRPA1, the results of which suggest that FIH-mediated hydroxylation may be linked to AITC-induced channel activation.

## Results

### TRPA1 binding to FIH involves the dimer interface

**Figure 1:**
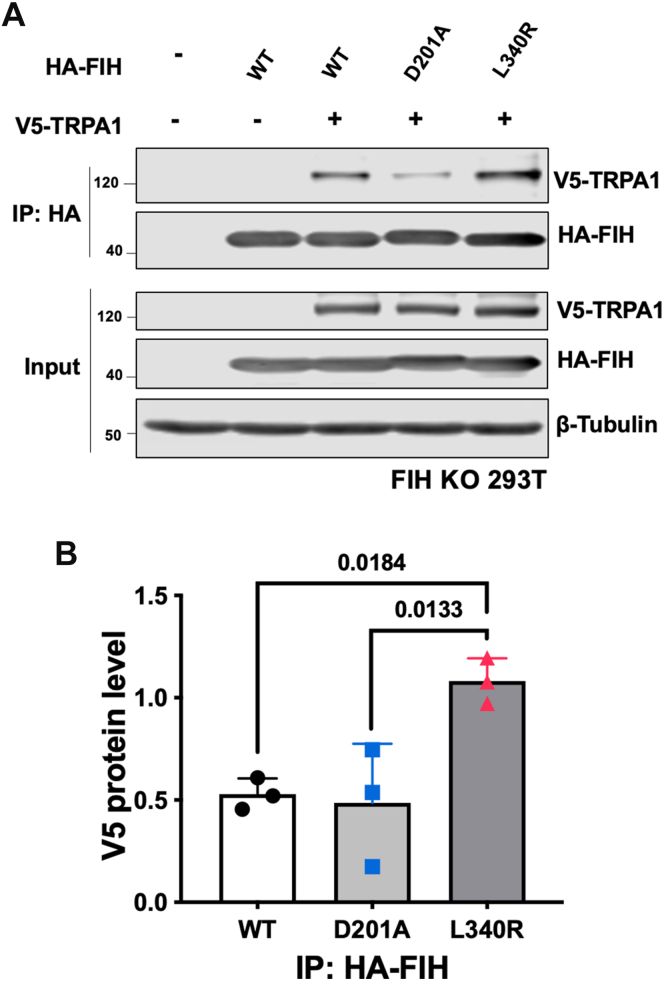
**TRPA1 binding to FIH involves the dimer interface.***A*, total cell lysates from FIH KO 293T cells transfected with the indicated plasmids were immunoprecipitated (IP) with an anti-HA antibody. V5-TRPA1 and HA-FIH levels are indicated. β-Tubulin was used as a loading control. *B*, graph showing protein levels of pulled-down V5-TRPA1 normalized to the total level of V5-TRPA1 in whole-cell lysates. Data are means ± SD with *p* values by Dunnett’s multiple comparisons test. FIH, factor-inhibiting hypoxia–inducible factor; HA, hemagglutinin; TRPA1, transient receptor potential cation channel, subfamily A, member 1.

We initially tested for interaction between FIH and TRPA1 using coimmunoprecipitation experiments. Genes encoding for WT hemagglutinin (HA)-tagged-FIH and HA-FIH variants with WT V5-TRPA1 were overexpressed in a human embryonic kidney (HEK) 293T cell “FIH CRISPR KO” subline (FIH KO 293T) with subsequent coprecipitation of HA-FIH. HA-FIH variants investigated included a catalytically inactive D201A variant and the L340R FIH variant, which, at least in some contexts, fails to dimerize (38). Interestingly, the L340R FIH variant showed increased coimmunoprecipitation of V5-TRPA1 compared with the WT FIH and D201A HA-FIH (Fig. 1; *p* < 0.05).

### Effects of FIH-mediated hydroxylation of TRPA1 on AITC-induced channel activation in TRPA1-expressing HEK 293T cells

**Figure 2:**
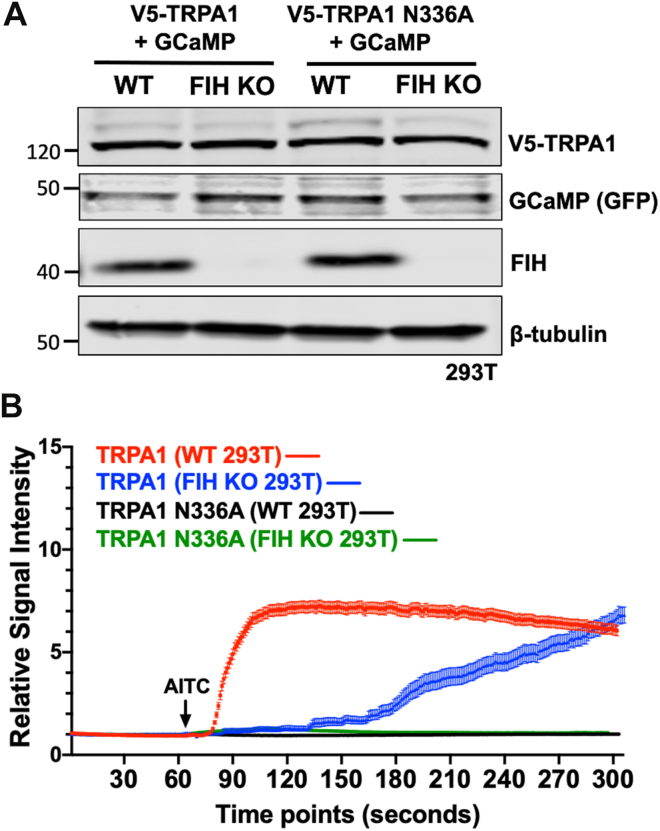
**Effects of FIH-mediated hydroxylation of TRPA1 on AITC-induced channel activation in TRPA1-expressed HEK 293T cells.***A*, total cell lysates from WT or FIH KO 293T cells transfected with the indicated plasmids were collected. V5-TRPA1, GCaMP (GFP), and FIH levels are indicated; β-tubulin was used as a loading control. *B*, time course of responses of WT or FIH KO 293T cells transfected with indicated plasmids to addition of AITC (20 μM) (*arrows* indicate addition points). Relative changes in GCaMP fluorescent levels are shown. Data are mean ± SEM (standard error of the mean). AITC, allyl isothiocyanate; FIH, factor-inhibiting hypoxia–inducible factor; HEK, human embryonic kidney cell line; TRPA1, transient receptor potential cation channel, subfamily A, member 1.

The TRPA1 agonist AITC increases intracellular calcium ion concentrations in cells expressing TRPA1 in a concentration-dependent manner (39). To investigate whether FIH-catalyzed hydroxylation of TRPA1 affects AITC-induced channel activation, WT and FIH KO HEK 293T cells were transfected with a vector encoding WT human TRPA1 and a fluorescent calcium sensor protein (GCaMP) (40) (Fig. 2*A*). We observed rapid changes in GCaMP fluorescence in WT TRPA1–expressing cells upon the application of AITC (Fig. 2*B*). Notably, a slow increase in GCaMP fluorescence was observed in FIH KO 293T cells expressing WT TRPA1 (Fig. 2*B*). To investigate the role of TRPA1 hydroxylation at N336, WT and FIH KO 293T cells were transfected with a vector encoding the hydroxylation-deficient TRPA1-N336A mutant (Fig. 2*A*). In each case, we observed that the increase in GCaMP fluorescence in response to AITC was eliminated (Fig. 2*B*). These observations suggest that FIH-mediated interaction with N336, including hydroxylation, may regulate TRPA1 sensitivity toward AITC.

### Effects of FIH inhibition on AITC-induced channel activation in murine hippocampal cultures

**Figure 3:**
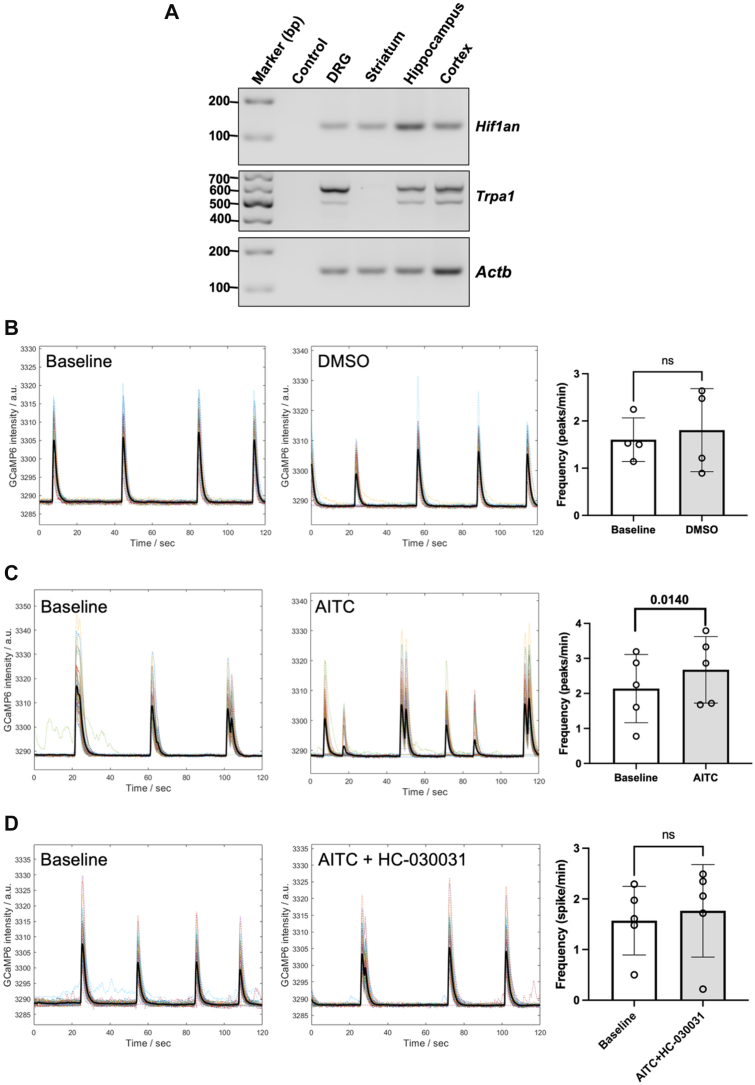
**Effects of DMSO and AITC in the absence or presence of HC-030031 on GCaMP fluorescence in murine hippocampal cultures.***A*, gene expression levels of FIH (*Hif1an*) and *Trpa1* in the indicated murine primary cultures. β-actin (*Actb*) was used as a loading control. *B*–*D*, representative traces and graphs showing GCaMP fluorescent levels in murine hippocampal cultures with the indicated treatment. AITC was applied at 20 μM, with 0.2% DMSO serving as the control. HC-030031, a selective and well-characterized TRPA1 antagonist, was used at 20 μM. Data are presented as mean ± SD, and statistical significance was assessed using a two-tailed paired *t* test. AITC, allyl isothiocyanate; DMSO, dimethyl sulfoxide; DRG, dorsal root ganglion; FIH, factor-inhibiting hypoxia–inducible factor; ns, not significant.

*TrpA1* is expressed in neurons of the central nervous system, including hippocampal cultures (Fig. 3*A*; Fig. 5*A*) (3), where it modulates neuronal activity (4). We detected FIH (*Hif1an*) mRNA expression in hippocampal, cortical, striatal, and dorsal root ganglia (DRG) cultures (Fig. 3*A*). The presence and functional role of Trpa1 in hippocampal cultures were further validated using AITC and HC-030031, a well-characterized and selective Trpa1 antagonist (41). Application of AITC, but not dimethyl sulfoxide (Fig. 3*B*), significantly increased neuronal activity (Fig. 3*C*; *p* = 0.0140), and this response was abolished by HC-030031 (Fig. 3*D*; *p* > 0.05).

**Figure 4:**
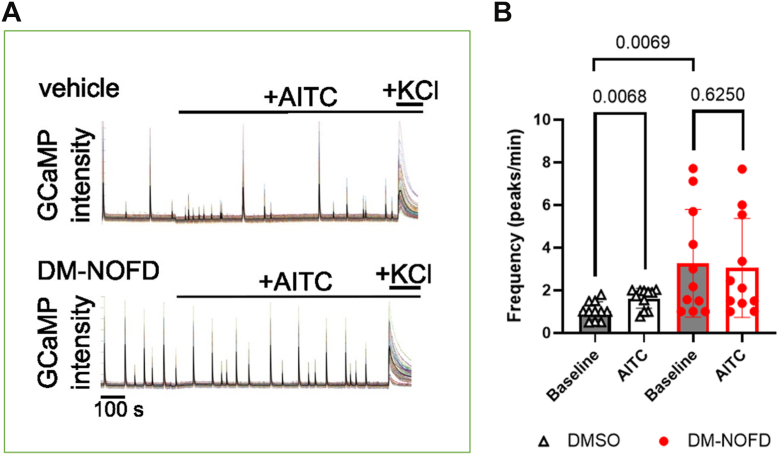
**Effects of FIH inhibition using DM-NOFD on AITC-induced channel activation in murine hippocampal cultures.***A*, representative traces of GCaMP fluorescent levels in murine hippocampal cultures with the indicated treatment. *B*, graph showing frequency (peaks/min) of GCaMP fluorescent levels in murine hippocampal cultures with the indicated treatment. AITC was applied at 20 μM. Data are mean ± SD with *p* values by Tukey’s multiple comparisons test. AITC, allyl isothiocyanate; DM-NOFD, dimethyl *N*-oxalyl-*D*-phenylalanine; FIH, factor-inhibiting hypoxia–inducible factor.

**Figure 5:**
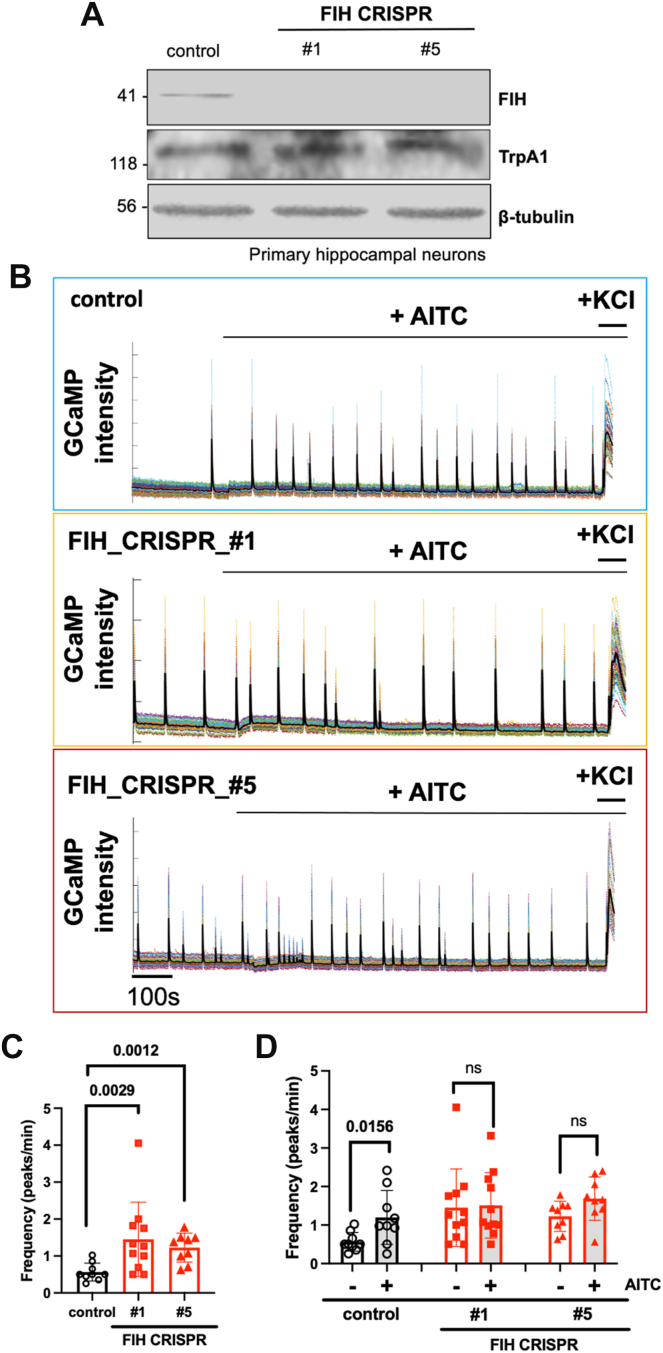
**Effects of FIH deletion using CRISPR gene editing on AITC-induced channel activation in murine hippocampal cultures.***A*, FIH and Trpa1 levels in total cell lysates from control or FIH-deleted murine primary hippocampal cultures using CRISPR gene editing; β-tubulin was used as a loading control. *B*, representative traces of GCaMP fluorescent levels in murine hippocampal cultures with the indicated treatment. *C* and *D*, graphs showing frequency (peaks/min) of GCaMP fluorescent levels in murine hippocampal cultures with the indicated treatment. AITC was applied at 20 μM. Data are mean ± SD with *p* values by Dunnett’s (*C*) or Tukey’s (*D*) multiple comparisons test. AITC, allyl isothiocyanate; FIH, factor–inhibiting hypoxia-inducible factor; ns, not significant.

To assess whether FIH affects neuronal activity in hippocampal cultures, we grew cells in the presence of a prodrug form of the FIH-selective inhibitor *N*-oxalyl-*D*-phenylalanine, that is dimethyl *N*-oxalyl-*D*-phenylalanine (DM-NOFD) (32, 42) for 14 days prior to calcium imaging as a readout of neuronal activity. Cultures grown with the inhibitor remain viable and indistinguishable by gross morphology from their untreated counterparts (Fig. S1). Growing cultures in the absence of active FIH increased the baseline frequency of Ca^2+^ transients (Fig. 4; *p* = 0.0069). Addition of AITC to activate Trpa1 leads to a significant increase in neuronal activity compared with vehicle-treated hippocampal cultures (Fig. 4; *p* = 0.0068). Under conditions of FIH inhibition (DM-NOFD), cultures showed an enhanced level of baseline excitability measured as GCaMP transient frequency. Trpa1 activation with AITC did not further increase the frequency of these Ca^2+^ transients when FIH was inhibited (Fig. 4; *p* = 0.6250). We obtained similar results using CRISPR-mediated knockout of FIH in primary hippocampal cultures. Two different guide RNAs efficiently targeted FIH in primary cultures (Fig. 5*A*). Both guides replicated the baseline increase in the frequency of Ca^2+^ transients observed with DM-NOFD (Fig. 5, *B* and *C*; *p* = 0.0029 and 0.0012, respectively). These cultures targeted for CRISPR-mediated FIH deletion also did not show a significant increase in neuronal activity in response to Trpa1 activation with AITC (Fig. 5, *B* and *D*; *p* > 0.05). Together, these observations suggest a physiological role for FIH and TRPA1 interaction in regulating neuronal activity patterns.

## Discussion

Cellular and functional analyses suggest that TRPA1's sensitivity to AITC is linked to FIH-mediated hydroxylation of TRPA1 N336. The lack of effect by AITC on neuronal cultures treated with FIH inhibitor or with CRISPR-mediated deletion suggests that hydroxylation of TRPA1 is required for discernible channel function. This aligns with our observations in TRPA1-expressing HEK 293T cells: both exhibit a clear response to AITC. However, this response is diminished or delayed in TRPA1-expressing HEK 293T cells and absent in primary hippocampal cultures when FIH activity is lacking. However, the mechanisms connecting asparagine hydroxylation to TRPA1 function remain unclear. Further analyses will be required to assess whether the lack of function results from reduced cell surface localization or impaired ion channel kinetics in nonhydroxylated TRPA1. The N-terminal cytosolic domain of TRPA1, which includes its ARD, is known to facilitate interactions with proteins such as calmodulin (43). Hydroxylation of asparagine residues within ARDs has been shown to stabilize the ARD fold (44), suggesting that TRP channel hydroxylation may influence the binding affinity of regulatory proteins. Phosphorylation of serine and threonine residues within the ARD has been reported to modulate TRPA1 sensitivity to AITC, likely by altering calcium influx (45). However, given the subtler nature of hydroxylation compared with phosphorylation, it may be considered unlikely that ARD hydroxylation will affect channel sensitivity through the same mechanism.

Interestingly, coimmunoprecipitation experiments imply that the L340R FIH variant, which is unable to efficiently dimerize (38), binds better with V5-TRPA1 than WT or catalytically impaired but dimeric D201A FIH (46). These observations suggest that TRPA1 binding to FIH may involve the FIH dimer interface. Crystallographic studies on FIH in complex with TRPA1 ARD fragments show active site cleft binding in a manner similar to that of HIF-1α and previously studied ARD FIH substrates (10). FIH forms a homodimer in solution, which can, at least in the crystal state, bind two substrates simultaneously (38). There is thus potential for the FIH dimer to simultaneously interact with two ARDs, from either the same or the different TRP channels. This potential for FIH to form dynamic complexes with two substrates that bind, but which may not both be hydroxylation substrates brings further complexity to the cellular biochemistry of FIH. In addition, FIH has been shown to catalyze hydroxylation of multiple residues (including asparagine, histidine, and aspartate residues) on a wide range of ARD-containing proteins (18, 19, 20, 21, 22, 23, 24, 47). Given the multiple cellular substrates of FIH, it is possible that some of the cellular observations linking FIH and TRPA1 channel function are indirectly mediated, potentially mediated by the effects of FIH on small-molecule redox-related metabolism (25, 26, 27, 28, 29). In future work, it will be important to show that FIH-catalyzed TRPA1 channel hydroxylation occurs under natural conditions; in this regard, development of antibodies selective for Asn-hydroxylated TRP channels is of interest.

Despite the potential complexities, inhibiting FIH, either through use of a selective inhibitor or CRISPR-mediated knockout in primary hippocampal cultures, resulted in an increased baseline frequency of Ca^2+^ transients. These findings demonstrate that FIH activity plays a key role in regulating neuronal network activity in cultured neurons, highlighting the significant impact of FIH activity on neuronal excitability—an essential process for sensory processing and cognition. The observed changes in baseline activity may result from directly inhibiting the hydroxylation of TRPA1 by FIH, but FIH inhibition is likely to affect other ARD proteins, including voltage-gated ion channels, scaffolding proteins, cytoskeletal components, or synaptic regulators involved in neuronal excitability. In addition, the increased frequency of Ca^2+^ transients may represent a form of homeostatic plasticity, wherein neurons adjust their intrinsic excitability to maintain stable network function. Furthermore, alterations in HIF signaling could influence oxygen homeostasis and metabolism, indirectly shaping neuronal activity. Further work is required to access these mechanisms.

We recognize that TRPA1 expression is robust in sensory ganglia (2) but is more controversial and likely lower in hippocampal neurons (3), which represents a limitation of our study. Nonetheless, our molecular and functional data support its presence and activity in hippocampal cultures, and even low-abundance ion channels can exert significant effects on network function. While DRG and trigeminal neurons remain the primary models for TRPA1 in nociception (2), hippocampal cultures provide complementary insight into potential central roles, such as stress responses and synaptic modulation. Future *in vivo* studies and cell-type–specific analyses will be needed to consolidate its contribution to neuronal physiology.

## Experimental procedures

### Cell culture and reagents

WT and FIH KO 293T cells, generously provided by Prof Sir Peter Ratcliffe and Dr Ya-Min Tian (University of Oxford), were cultured in Dulbecco's modified Eagle's medium Invitrogen), supplied with 10% fetal bovine serum (Sigma–Aldrich) and antibiotics as appropriate. All cells were cultured at 37 °C and 5% CO_2_. Cells were transfected with the indicated plasmids using the Lipofectamine 3000 Transfection Reagent (Invitrogen) according to the manufacturer’s instructions.

All experiments using primary murine cultures were performed in accordance with the Animals (Scientific Procedures) Act 1986 set out by the UK Home Office and approved by the University of Southampton Ethics Committee. For primary neuronal cultures, required brain regions or ganglia were isolated from E15–E18 C57BL/6 embryos and dissociated with 0.05% trypsin for 6 to 8 min at 37 °C before quenching the trypsin with fetal bovine serum. The solution was exchanged for culture medium (2% B27 supplement in phenol red–free neurobasal medium, 0.5 mM GlutaMax), and cells were dissociated by carefully pipetting six to eight times. Cells were plated on poly-d-lysine–coated dishes. For DRG cultures, media were further supplemented with 100 ng/ml nerve growth factor. Neuronal cultures were transduced at day *in vitro* 1 using the indicated lentivirus (details provided in the Supporting Information). HC-030031 was from MedChemExpress (HY-15064).

### Immunoprecipitation and Western blot analysis

Western blot analyses were performed with lysates from cells lysed with urea buffer (8 M urea, 1 M thiourea, 0.5% CHAPS, 50 mM DTT, and 24 mM spermine). The bound proteins were separated on SDS polyacrylamide gels and subjected to immunoblotting with the indicated antibodies. For immunoprecipitations, cells were lysed for 30 min at 4 °C in 50 mM Tris–HCl (pH 7.5), 120 mM NaCl, 1 mM EDTA, and 0.1% Nonidet P-40 detergent (Sigma–Aldrich), with protease inhibitors (cOmplete, EDTA-free Protease Inhibitor Cocktail; Roche). Anti-HA (Santa Cruz Biotechnology; sc-7392) or control antibodies and Protein G magnetic beads (Thermo Fisher Scientific) were added to the lysate for 16 h at 4 °C. Immunoprecipitates were washed four times with cold PBS followed by the addition of SDS sample buffer. The bound proteins were separated on SDS polyacrylamide gels and subjected to immunoblotting with the indicated antibodies. Primary antibodies were from Cell Signaling Technology (β-tubulin, 1:5000 dilution, 86298, mouse monoclonal; HA, 1:1000 dilution, 3724, rabbit monoclonal), Santa Cruz Biotechnology (GFP, 1:500 dilution, sc-9996, mouse monoclonal), Moravian Biotech (murine FIH, clone 3F9, 1:25; kindly provided by Prof Xin Lu, University of Oxford) (35), human FIH (monoclonal 162 C; kindly provided by Prof Sir Peter Ratcliffe, University of Oxford), Bio-Rad Laboratories (V5, 1:1000 dilution, MCA1360, mouse monoclonal), and Novus Biologicals (TRPA1, 1:500 dilution, NB110-40763, rabbit polyclonal). Signals were detected using an Odyssey imaging system (LI-COR) and evaluated by ImageJ 1.42q software (National Institutes of Health). Uncropped Western blot images are provided in the Supporting Information.

### RT–PCR

RNA was isolated from primary cultures using the RNeasy kit (QIAGEN), and 200 ng of RNA were reverse transcribed using oligo-dT primers and the Precision nanoScript 2 reverse transcription kit (Primer Design) according to the manufacturer's instructions. The resulting complementary DNA was analyzed by end-point PCR using RedTaq ReadyMix PCR Reaction Mix (Sigma–Aldrich) and a GeneAmp PCR System 9700 (Applied Biosystems). The primers and PCR conditions are listed in Table S1. Uncropped agarose gel images can be found in the Supporting Information.

### Live-cell imaging and analysis

Cells were plated on glass-bottom dishes (WillCo Wells) and transfected with the indicated plasmids using Lipofectamine 3000 Transfection Reagent (Invitrogen) according to the manufacturer’s instructions. GCaMP6s was the gift from the Douglas Kim & GENIE Project (Addgene plasmid #40753; Research Resource Identifier: Addgene_40753). Prior to imaging, cells were switched into 1 ml phenol red–free Dulbecco's modified Eagle's medium supplemented with 20 mM Hepes–NaOH (pH 7.4) and imaged using an inverted fluorescence DeltaVision Elite microscope equipped with a temperature-controlled chamber maintained at 37 °C, 20x UPlanSApo objective (numerical aperture = 0.75), SSI 7-band LED, UltimateFocus and a monochrome sCMOS camera, using SoftWoRks software v6. Images were taken every second for 5 min. After sampling the baseline fluorescence for 60 s, 1 ml (2x final concentration, 20 μM, in imaging media) AITC was added to the dish to allow immediate mixing while continuing acquisition for a further 4 min. As a control, 0.2% dimethyl sulfoxide was used. HC-030031 (MedChemExpress, HY-15064), a well-characterized and selective TRPA1 antagonist, was used at 20 μM. Images were analyzed by outlining cells in the final frames of the movie, measuring fluorescence intensity across all frames in the stack using ImageJ (National Institutes of Health) and expressing it as F/F0.

Murine hippocampal neuronal cultures were transduced using lentivirus and imaged at days *in vitro* 13 to 15. Images were taken every 200 ms as described above. Relative frequency was analyzed as reported (48).

### Statistical analysis and repeatability of experiments

Statistical analyses were performed using R (version 4.3.0) or GraphPad Prism, version 10 (GraphPad Software, Inc). For primary cultures, imaging data were derived from at least four independent preparations, and each data point relates to one plate.

Normality was evaluated using the D’Agostino–Pearson test. For two-group comparisons, Student’s *t* test was applied for parametric data, whereas the Mann–Whitney *U* test was used for nonparametric data. Multiple parametric comparisons were analyzed using one- or two-way ANOVA followed by Tukey’s post hoc test, whereas the Kruskal–Wallis test with Dunnett’s post hoc test was utilized for nonparametric comparisons. Results were considered significant if *p* < 0.05.

## Data availability

The authors confirm that the data supporting the findings of this study are available within the article and its supporting information.

## Supporting information

This article contains supporting information.

## Conflict of interest

The authors declare that they have no conflicts of interest with the contents of this article.
