# Supplementary material for: Cellular and functional insights into FIH-mediated hydroxylation of TRPA1
Source: J Biol Chem. 2025 Nov 4;301(12):110882. doi: 10.1016/j.jbc.2025.110882 (PMC12719648; doi:10.1016/j.jbc.2025.110882)
Supplement: Supplementary Materials [file mmc1.pdf]

Supplementary Materials

Cellular and functional insights into FIH-mediated hydroxylation of TRPA1

Tao Guo[1,#], Dianne Marquez Lopez[1,7,#], Siyuan Wang[1,#], Liudi Yao[1], Xi Li[1], Elizabeth R. Davies[1,2], Mariana Vargas-Caballero[1,3], Nullin Divecha[1,3], Christopher J. Schofield[4], Katrin Deinhardt[1,3,5]*, and Yihua Wang[1,3,6]*

[1]Biological Sciences, Faculty of Environmental and Life Sciences, University of Southampton, Southampton SO17 1BJ, U.K.

[2]Clinical and Experimental Sciences, Faculty of Medicine, University of Southampton, Southampton SO16 6YD, U.K.

[3]Institute for Life Sciences, University of Southampton, Southampton SO17 1BJ, UK.

[4]Department of Chemistry and the Ineos Oxford Institute for Antimicrobial Research, Chemistry Research Laboratory, Mansfield Road, University of Oxford, Oxford OX1 3TA, U.K.

[5]University of Bremen, Cell Biology, 28359 Bremen, Germany

[6]NIHR Southampton Biomedical Research Centre, University Hospital Southampton, Southampton SO16 6YD, U.K.

[7]UK Dementia Research Institute, Centre for Discovery Brain Sciences, University of Edinburgh, Edinburgh Bioquarter, Edinburgh EH16 4SB, U.K.

Supplementary Methods

FIH deletion using CRISPR gene editing in murine hippocampal neurons

1. CRISPR-Cas9 plasmid design and construction

Single-guide RNAs (sgRNAs) targeting the mouse FIH (*Hif1an*) gene (Ensembl Gene ID: ENSMUSG00000036450) were designed using the CRISPick online tool from the Broad Institute (https://portals.broadinstitute.org/gppx/crispick/public). The gRNA sequences shown below were cloned into lentiCRISPR v2 (Addgene #52961). After sequence verification, the plasmids were prepared for subsequent experiments.

Oligo name	Sequence 5'-3'	Exon
FIH CRISPR #1	TAGAGGCACTCGAACTGATC	4
FIH CRISPR #5	GGTCACTCTGGCTCAGACGT	1

2. Preparation of lentiviral particles

Lentiviral particles were generated by transfecting HEK293FT cells with either empty or FIH-targeting lentiCRISPR v2 plasmids, along with the packaging plasmids pVSV-G (Addgene #8454) and pGag-Pol (Addgene #12260). The transfection mixture was prepared in a sterile Eppendorf tube as detailed below for each well of a 6-well plate and incubated at room temperature for 15 minutes before being added to the HEK293FT cells

Component	Volume or Amount
Opti-MEM	500 µL
lentiCRISPR	1 µg
pVSV-G	0.5 µg
pGag-Pol	1 µg
PEI (Polyethylenimine, 1mg/ml)	7.5 µL

The media were replaced 12 hours after transfection, and viral supernatants were collected at 24- and 48-hours post-replacement. The supernatant was collected in DMEM containing 10% FBS. Viral particles were purified via ultracentrifugation, with virus-containing supernatants aliquoted into 1.5 mL ultracentrifuge tubes and centrifuged at 100,000 × g for 1 hour and 20 minutes at 4 °C. After centrifugation, the supernatant was carefully removed, and the viral pellets were resuspended in serum-free DMEM to achieve a final concentration of 15×. The suspension was thoroughly mixed and incubated at room temperature for 4 hours to ensure complete dissolution. Finally, the virus-containing solution was passed through a 0.45 μm filter to remove debris, yielding the purified virus preparation.

3. Lentiviral transduction of target cells

Primary murine hippocampal neurons were seeded onto coated glass-bottom plates (Mattek, P35G-1.5-20-C) and allowed to adhere overnight. The next day (DIV1, day *in vitro* 1), each dish was transduced with 150 μL of 15× lentiviral particles expressing GCaMP6 and 250 μL of 15× either empty or FIH-targeting lentiCRISPR v2 plasmids, followed by a 24-hour incubation. The medium was then refreshed with 1 mL of fresh Neurobasal medium (supplemented with B27 and GlutaMAX) and 1 mL of conditioned medium to prepare for subsequent assays.

Supplementary Figures

Supplementary Figure 1. Representative hippocampal neuron cultures.
Hippocampal neurons were treated with a FIH inhibitor, 1 mM DM-NOFD, or vehicle control (DMSO), every 3-4 days and images were taken at indicated time points. Scale bar = 100 µm. DIV: day *in vitro*.

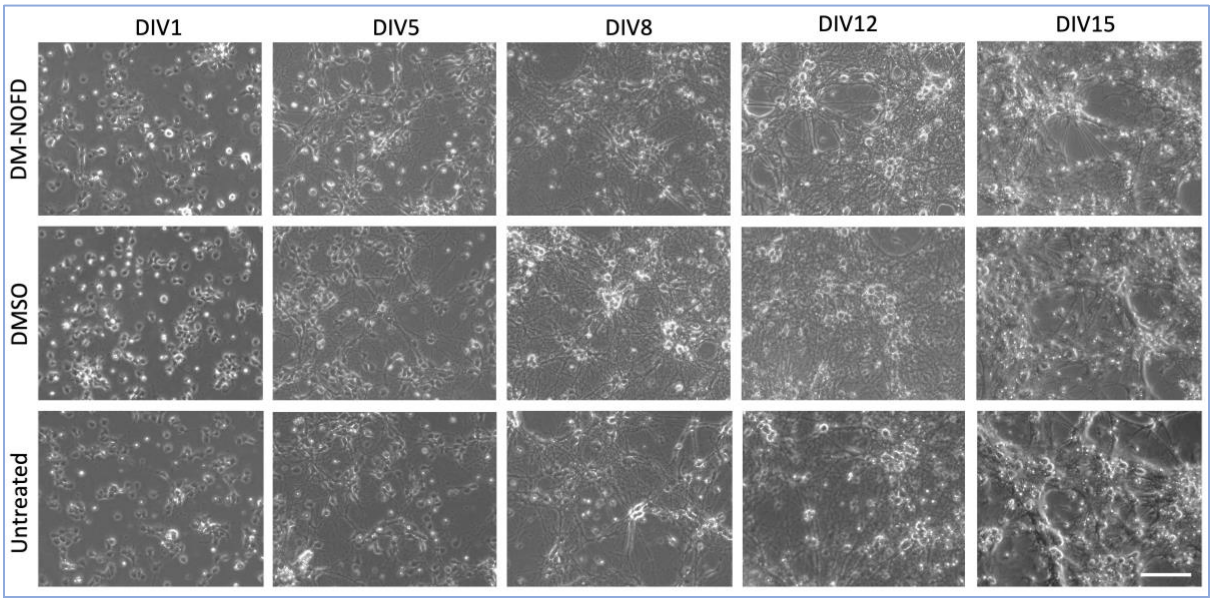

Supplementary Tables

Supplementary Table 1 - RT-PCR primers and conditions

Gene	Forward/Reverse	Primer sequence	Expected amplicon sizes	End-point-PCR Tm (°C)	Brand	Cat. no
Ank3	Forward	CTGCCTCCACTACAGCCTCT	174	58	Eurofin	//
	Reverse	GGAGAGAAGCCTGAGCAGAA				
Trpa1	Forward	AACTCCTCAACCACCCTGTG	577, 487	58	Eurofin	//
	Reverse	CTGAGGCCAAAAGCCAGTAG				
Hif1an	Forward	GGCTACTTTCTGATGAGCTT	138	55	Eurofin	//
	Reverse	CAATGTACTGGTGGCACCATATAG				
Actb	Forward	//	149	55	Qiagen	QT00095242
	Reverse	//				

Tm: melting temperature

Uncropped western blot and agarose gel images -

Figure 1A

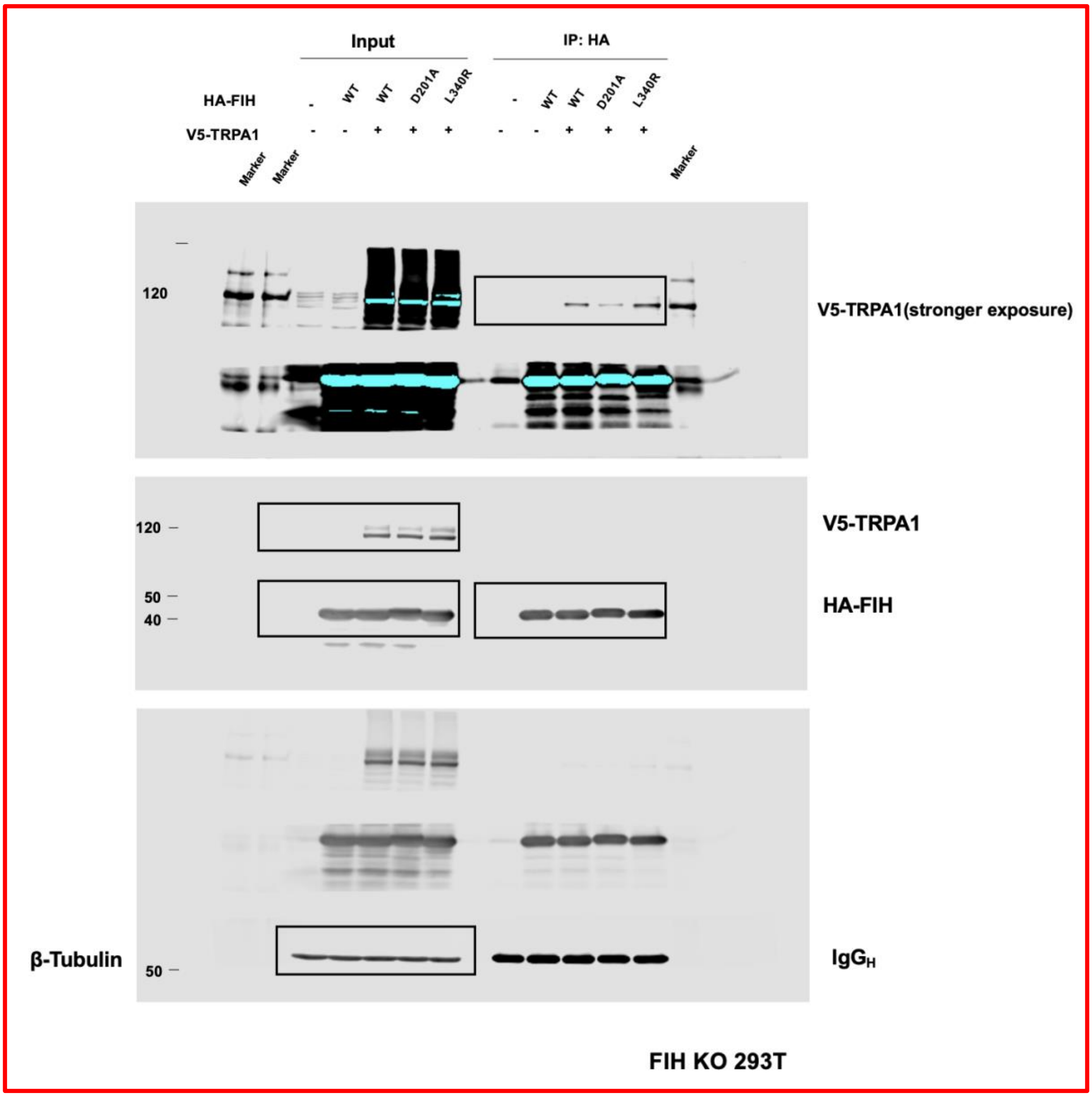

Figure 2A

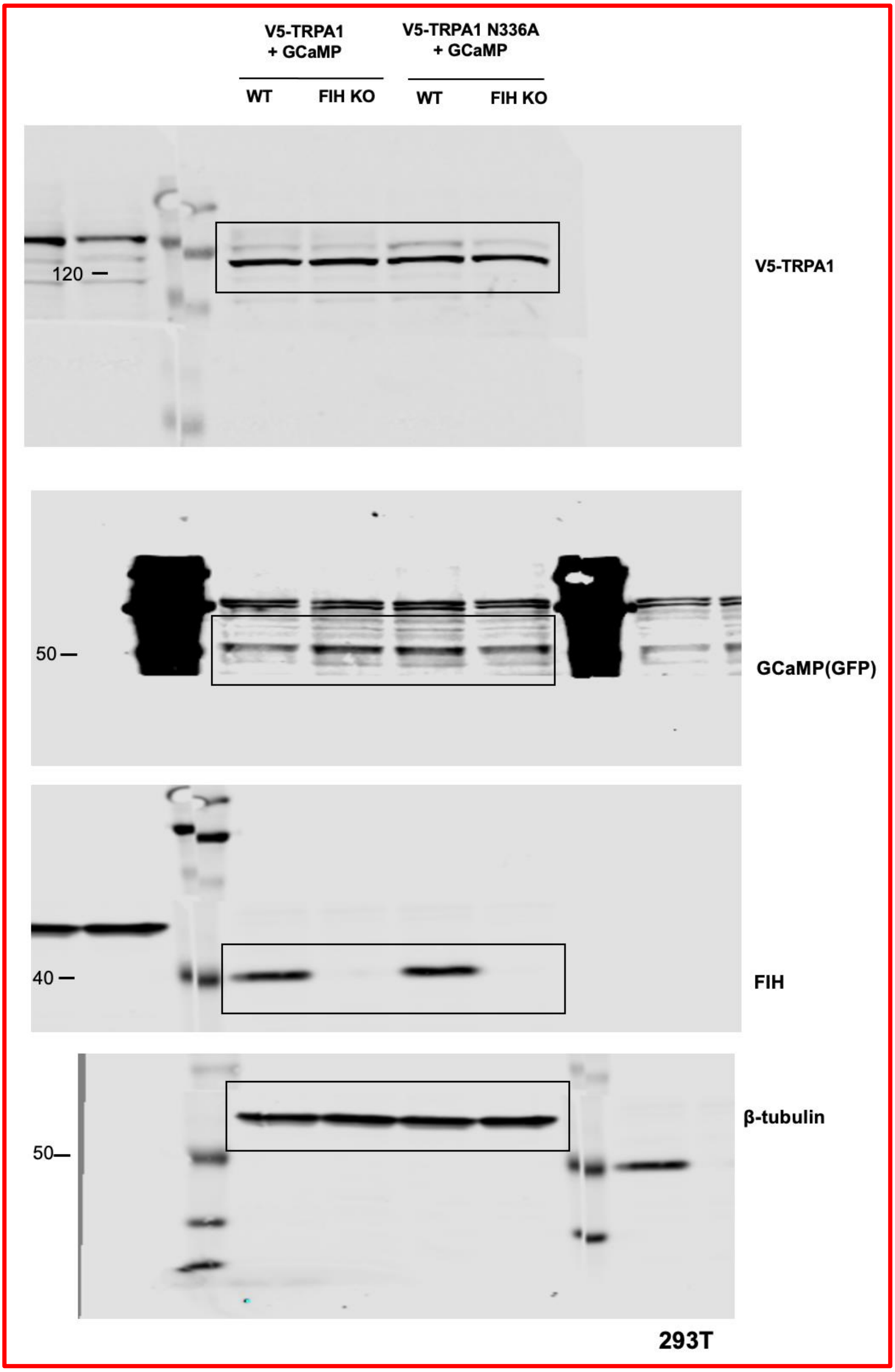
V5-TRPA1
+ GCaMP
V5-TRPA1 N336A
+ GCaMP
WT
FIH KO
WT
FIH KO
120
V5-TRPA1
50
GCaMP(GFP)
40
FIH
β-tubulin
50
293T

Figure 3A

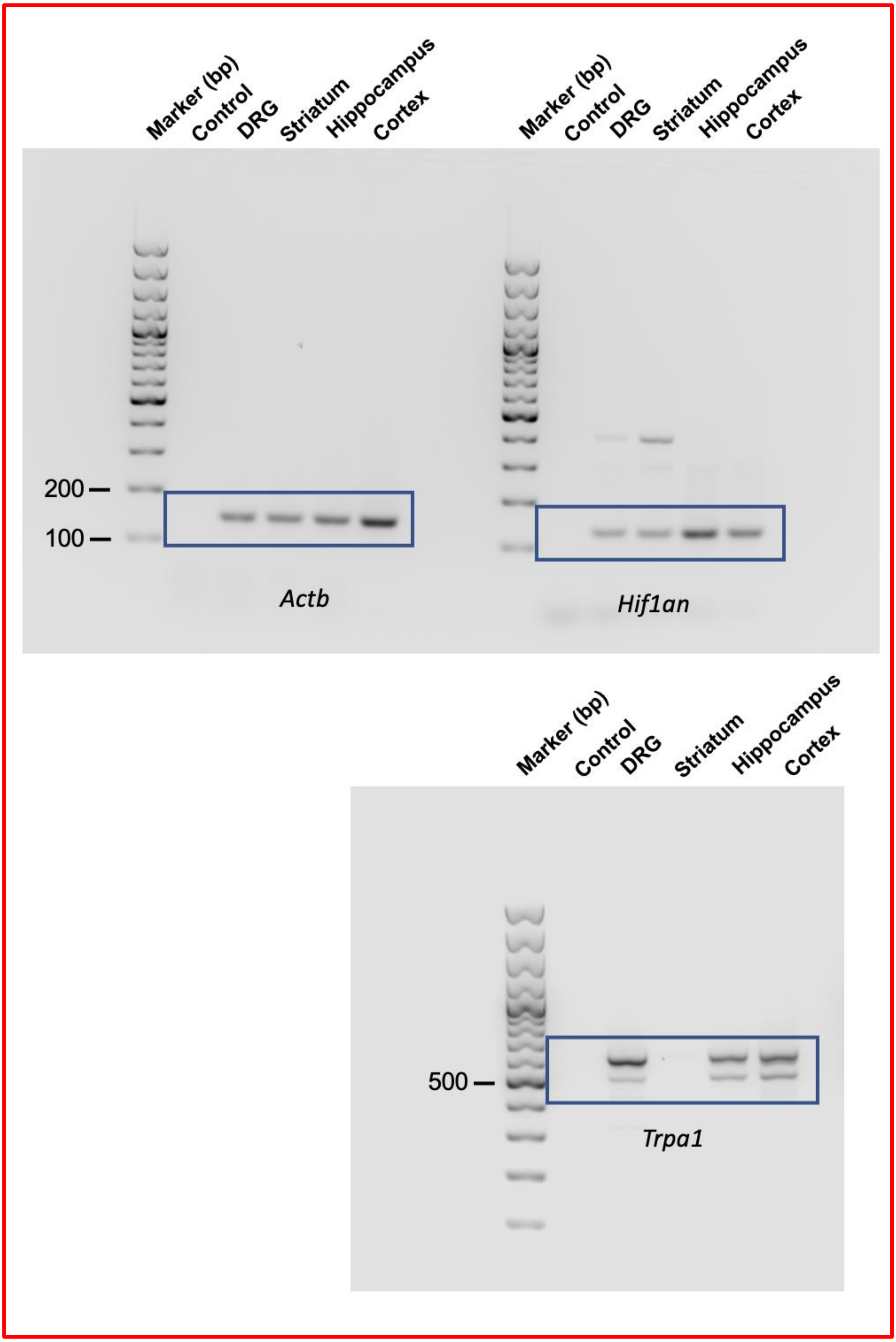
Marker (bp)
Control
DRG
Striatum
Hippocampus
Cortex
200
100
Actb
Marker (bp)
Control
DRG
Striatum
Hippocampus
Cortex
Hif1an
Marker (bp)
Control
DRG
Striatum
Hippocampus
Cortex
500
Trpa1

Figure 5A

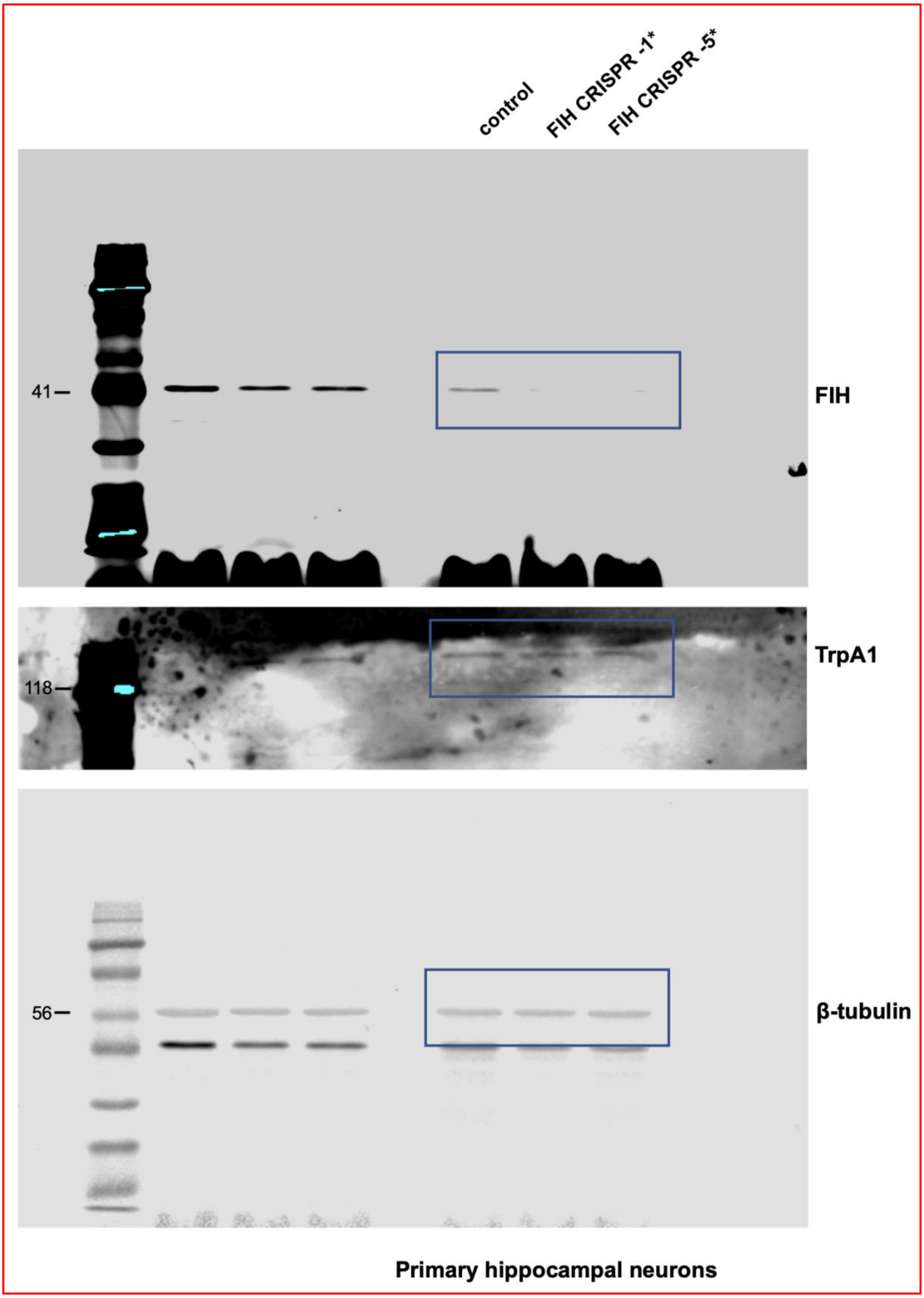
control
FIH CRISPR -1*
FIH CRISPR -5*
41
FIH
118
TrpA1
56
β-tubulin
Primary hippocampal neurons
